# Lab-on-PCB with integrated DNA amplification and electroanalytical detection for point-of-care diagnostics

**DOI:** 10.1038/s41598-025-12364-1

**Published:** 2025-09-12

**Authors:** Seshagopalan Thorapalli Muralidharan, Martin Hanze, Alar Ainla, Björn Möller, Mahiar Max Hamedi, Anna Toldrà

**Affiliations:** 1 Department of Fiber and Polymer Technology, KTH Royal Institute of Technology, Teknikringen 56, 10044 Stockholm, Sweden; 2 Department of Engineering Design, KTH Royal Institute of Technology, Brinellvägen 83, 10044 Stockholm, Sweden; 3 International Iberian Nanotechnology Laboratory, 4715-330 Braga, Portugal; 4 SciLifeLab, Department of Microbiology, Tumor and Cell Biology, Karolinska Institute, Tomtebodavägen 23A, 17165 Solna, Sweden

**Keywords:** Printed circuit board (PCB), Lab-on-PCB., Nucleic acid amplification test (NAAT)., Point-of-care., Electrochemical biosensor., Electrical and electronic engineering, Biochemical assays, Hardware and infrastructure

## Abstract

Nucleic acid amplification tests (NAATs) are powerful medical diagnostic tools for point-of-care (POC) and other field applications. However, traditional methods like quantitative PCR (qPCR) require complex, expensive equipment and trained operators, limiting their use to centralized labs. Isothermal alternatives, like Loop-mediated Isothermal Amplification (LAMP), are better adapted for POC devices. Lab-on-PCB systems have the potential to overcome the challenges faced by conventional microfabrication-based systems. This study presents a novel lab-on-PCB device for nucleic acid amplification and electrochemical detection using reverse transcription LAMP (RT-LAMP) of SARS-CoV-2. The system consists of two disposable PCB-based chips making it close to zero cost. One PCB is for heating and nucleic acid amplification, while the other is for electrochemical detection using Cyclic Voltammetry (CV) with a redox-active intercalator. The PCB slides are connected to a compact electronic device (< 10 USD) for controlling the heating and electroanalytical readout. Using this device, we achieved successful rapid (< 1.5 h) nucleic acid amplification and detection at a target concentration of 10 copies/reaction. This work represents a notable step toward developing integrated, portable NAAT devices for POC diagnostics.

**Supplementary Information:**

The online version contains supplementary material available at 10.1038/s41598-025-12364-1.

## Introduction

Nucleic acid amplification tests (NAATs) are a set of techniques used to amplify and detect nucleic acids from a sample, used in medical diagnostics and health surveillance, as well as in fields such as food safety, forensics, and environmental monitoring. These techniques are highly sensitive and specific, enabling the identification of genetic material even at low concentrations^1,2^. Quantitative Polymerase Chain Reaction (qPCR) is the gold NAAT and it requires precise cycling and control of temperatures to maintain the reaction. In practice, this is performed using benchtop and generally expensive instruments called thermocyclers, which also contain optical units for the readout^3^.

Due to the complexity of the steps required in NAATs, specialists are needed to operate these instruments. Therefore, NAATs are generally limited to centralized facilities such as hospital laboratories. This creates a delay between sampling and result, which especially in the case of diagnosing infectious diseases could negatively affect the patient outcome^4^. Much work has been put into developing point-of-care (POC) diagnostic devices that can be used at or near the site of the patient, including in remote, resource-limited settings^5^. Isothermal NAATs such as Loop-mediated Isothermal Amplification (LAMP) promises easier integration into such devices^6^. Amplification-free approaches have also been proposed^7–9^, and while these approaches promise reduced complexity, amplification offers high specificity using target-complementary primers and innate signal amplification.

The concept of Lab-on-a-Chip microfluidic devices, manufactured using standard micro-electromechanical system technologies, was proposed early on as a means to realize micro total analysis systems (µTAS)^10–14^ but so far there have been few commercial successes, partly because of the costs involved in cleanroom manufacturing and due to higher startup costs for microfluidic device manufacturing^15,16^. Cheaper alternative technological platforms include paper^17–21^ or textile-based^22–29^ microfluidic and sensing platforms. Another option that has not been as explored as much but is significantly cheaper than microsystems - yet retains a similar level of reproducibility, robustness, and ease of mass-manufacturing - is using the well-established and standardized Printed Circuit Board (PCB) technology^30^. The concept of Lab-on-PCB was proposed in the 1990s^30^ yet it has not received much attention as Lab-on-a-Chip. But this trend could be changing^31–35^; there have been multiple attempts at making PCB-based, portable thermocyclers for PCR tests^34–44^. There has, however, been to our knowledge no PCB-based totally integrated NAAT test, where the amplification and electroanalytical detections are integrated into a single low-cost platform.

We have previously shown a type of disposable electrochemical PCB sensor that fits into a standard 200 µL microcentrifuge tube. Through this we were able to perform a sandwich hybridization assay on isothermally amplified DNA by sequentially dipping the device in a series of tubes. The electrochemical readout was achieved by connecting the PCB sensor to a portable potentiostat. This system demonstrates the potential of utilizing commercial PCBs as a technology for both the electrode and the driver electronics in electroanalytical nucleic acid detection^45^.

**Fig. 1:**
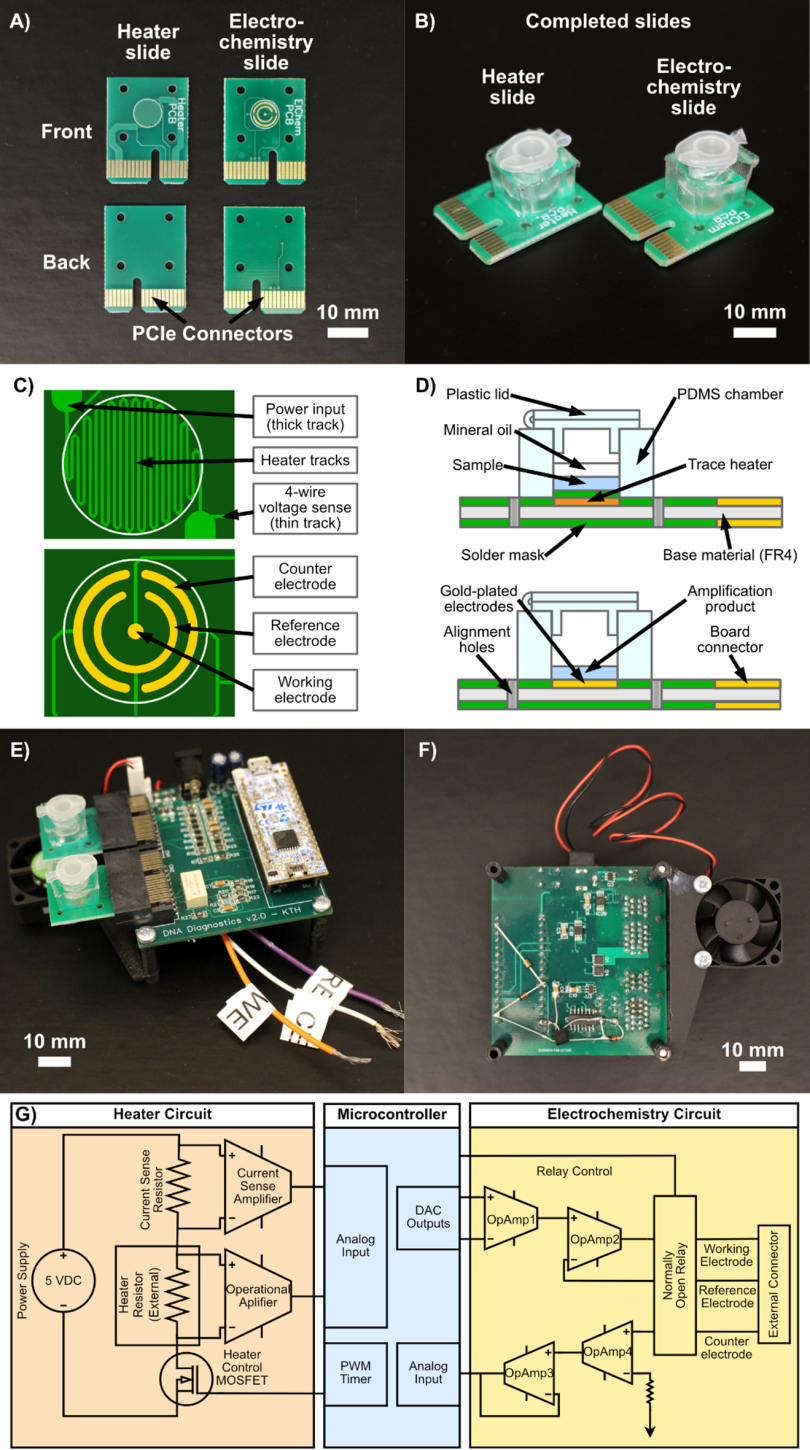
Overview of the Lab-on-PCB system. **(A)** A photograph showing the front and back of the heater PCB slide and the electrochemistry PCB slide. **(B)** A photograph showing the completed PCB slides with attached PDMS chambers and plastic lids. **(C)** Schematics of the copper trace coil heater of the heater PCB slide (top) and the electrode surfaces of the electrochemistry PCB slide (bottom). **(D)** An annotated sideway cross-section schematic of the heater PCB slide (top) and electrochemistry PCB slide (bottom). **(E)** A photograph of the main unit with both PCB slides inserted in the connector slots. **(F)** Photographs of the bottom side of the main PCB unit. Note that the cables used to connect to the conventional potentiostat has been removed for clearer view of the board. **(G)** A visual overview of the circuitry of the main unit.

Here we considerably advanced the NAAT devices by presenting a new approach toward Lab-on-PCB devices by integrating controlled heaters for isothermal NAATs^46–50^ as well as electrodes directly onto the PCB with no external components. This novel device uses two types of disposable PCB slides with polydimethylsiloxane (PDMS) chambers (Fig. 1A and B); one with a heating surface for performing reverse transcription LAMP (RT-LAMP) on a synthetic RNA sequence from SARS-CoV-2 and the other one containing a gold-printed three-electrode setup for electrochemical detection of the RT-LAMP product using Methylene Blue (MB) as an immobilization-free^51^ redox-active intercalator, which reduces the manual steps involved in the entire test. These slides can be inserted into a main unit (Fig. 1E and F), which is PCB-based with a microcontroller, that controls the amplification protocol and acts as a potentiostat for electrochemical detection. We have also developed a MATLAB Simulink-based computer software that can control the device. This device is compact, portable, and based on PCB technology which allows it to be produced at low cost at both small and large scale. The novelty in our system lies in the combination of isothermal amplification and electroanalytical readout using PCBs. Previously reported devices often rely on fluorescent detection, which requires the incorporation of relatively expensive and/or large components such as advanced optics, adding complexity and hindering scalability. It is also very likely that our system could be adapted for real-time detection in the future, and it can easily be expanded to carry out many tests at once with negligible increase in cost or portability.

## Experimental

### Materials

Molecular grade nuclease-free water, Methylene Blue (MB), potassium hydroxide, sodium sulfate, potassium chloride, and ferricyanide were purchased from Sigma-Aldrich (Stockholm, Sweden). Hydrogen peroxide 30%, TBE buffer, GelRed stain, TopVision Agarose, and GeneRuler 50 bp DNA Ladder were purchased from Thermo Fisher (Stockholm, Sweden). PDMS (Dow Sylgard 184) was purchased from GA Lindberg Chemtech (Stockholm, Sweden). WarmStart^®^ LAMP Kit (DNA & RNA) (New England Biolabs) was purchased from BioNordika (Solna, Sweden). Customized DNA oligonucleotides were synthesized by Biomers (Ulm, Germany). Table S1 shows all oligonucleotide sequences. All components mounted on the PCBs were purchased from Mouser Electronics (Malmö, Sweden), and the temperature probes were purchased from RS Components (Gothenburg, Sweden).

### PCB design and fabrication

Our Lab-on-PCB system consisted of three PCBs: the main unit, the heater slide, and the electrochemistry slide. These PCBs are described in detail in the following paragraphs. The PCBs were designed using EasyEDA and were manufactured by JLCPCB (China). The thickness of all PCBs was 1.6 mm. The main PCB was a 4-layer board, and the heater and electrochemistry PCBs were 2-layer boards. In the main PCB, the outer copper layers were 1 oz/ft^2^ and the inner copper layers were 0.5 oz/ft^2^. The electrochemistry and heater PCBs were ordered in a 3 × 4 panel. Both electrochemistry and heater PCBs were surface treated with the ENIG (Electroless Nickel, Immersion Gold) process, ensuring that the exposed layers of the board contained a layer of gold on top. In this case, the thickness of the gold coating was 0.0254 μm (1 micro inch).

#### Main unit

Detailed circuit schematics of the main unit are shown in Figures S1, S2, and S3. The circuitry consisted of the following three sections:

*1) Microcontroller module:* This section consisted of a microcontroller that was responsible for the orchestration and control of the heating and electrochemistry functions. The microcontroller was also responsible for communication with an external control PC. We used an STM32 Nucelo-G431KB development board with an STM32G431KB integrated circuit (IC) microcontroller running at 170 MHz, with 128 kb of flash, two 12-bit integrated ADC, a 12-bit two-channel DAC, and multiple PWM outputs. The microcontroller was connected to the other modules of the main PCB and connected to a computer over USB.

*2) Heater circuit module:* This section consisted of two sub-circuits with similar designs: the main heater and the air heater. While the main heater was used in the heater PCB slide, the air heater was implemented for use in a future circuit and remained unused in this work. The main heater circuit achieved sample heating by supplying power to a heater coil patterned in the copper trace of the PCB. Additionally, the resistance of this coil was monitored by measuring the current supplied to the coil and by measuring the voltage drop across the coil. This smart method allowed us to circumvent the use of any other components such as temperature sensors, thus reducing the cost, complexity, and sustainability of the one-time-use chips

The main heater section relied on three sub-modules, the MOSFET amplifier section, the voltage measurement section, and the current measurement section. The MOSFET amplifier section consisted of a low-side MOSFET manufactured by Vishay with the part number SISA18ADN-T1-GE3. This MOSFET was controlled using PWM signals supplied by the microcontroller. The MOSFET and associated circuitry were designed to supply up to 5 A current. The MOSFET consisted of a series gate resistor of 100 Ω and a gate-source resistor of 10 kΩ. The voltage measurement section consisted of a differential amplifier that was implemented using the Texas Instruments OPA2991IDGKR amplifier. The differential amplifier was set up to measure the 4-wire Kelvin resistance of the heater coil. The gain of the differential amplifier was 1. The circuit in this case used a MOSFET to switch the current. Therefore, the output of the amplifier was connected to an RC low-pass filter. This RC low pass filter was used to filter out the switching signal. The − 3 dB cutoff frequency of the low-pass filter was 159.1549 Hz. The current measurement section used a high-side shunt resistor of 0.01 Ω and a current sense amplifier to measure the series current in the circuit. A low-value resistor was adopted due to its small volume and low power loss. The current measurement was implemented by measuring the voltage drop across the shunt resistor. Since the voltage drop was small, a current sense amplifier was used to amplify the voltage drop. The current sense amplifier was a Texas Instruments INA2180A2IDGKT with a gain of 50 V/V. Like the voltage amplifier, the output of the current sense amplifier was connected to an RC low-pass filter with a −3dB cutoff frequency of 159.1549 Hz.

*3) Electrochemistry circuit module:* This section was designed similarly to the portable potentiostat that we have previously reported^42^. The operation needed to trigger the electrochemistry consisted of applying a varying potential on the counter electrode with respect to the pseudo-reference electrode. The potential on the counter electrode was determined by the profile of the test being conducted.

The electrochemistry (potentiostat) circuit consisted of two sub-modules, one maintains the set electrode potential and the other uses trans-impedance amplifier to measure current. In the potential setting stage, a potential was applied on the counter electrode with respect to the pseudo-reference electrode. The potential applied was decided by the profile. The potential setting stage consisted of two operational amplifiers. The first operational amplifier was configured as a differential amplifier, with the inputs as the DAC channels from the microcontroller. The output of the differential amplifier was computed using the following Eq. 1:

1 $$V_{{out}} = - 0.196.ch2DAC + 0.7984.ch1DAC$$

where *V*_*out*_ is the output voltage of the differential amplifier network used to set a voltage on the counter electrode, and *ch1DAC* and *ch2DAC* are the output voltages on channels 1 and 2 of the internal DAC, respectively. The output of the differential amplifier was connected to the non-inverting input of the second operational amplifier and the inverting input of the second operational amplifier was connected to the pseudo-reference electrode. A voltage on the counter electrode could therefore be set by varying the outputs on the DAC channels. Since the maximum possible DAC output from the microcontroller is 3.3 V, the range of output voltage is from − 0.6468 V to 2.60502 V. A least count of 0.8mV can be achieved, however considering a 1-bit error, a realistic least count of 1.61mV is achieved.

The other sub-section, the current feedback stage, consisted of two operational amplifiers. The operational amplifiers for all stages were made of the Texas Instruments LM2902 amplifiers. The first stage of the amplifier was designed as an inverting amplifier with a high gain like a transimpedance amplifier. The output of this stage was connected to a follower amplifier to ensure low output impedance. The output of this stage was clamped to 3.3 V and ground respectively using diodes.

#### Heater slide

The heater PCB was designed as a trace heater utilizing the heating traces designed on the copper-clad PCB (see Figure S4 for CAD drawings). This design was developed for a manufacturing process with a minimum trace width of 6 mil (0.152 mm) with a track spacing of 6 mil. To maximize the heat transfer, a circular outer contour with an outer diameter of 7 mm was used. To fit multiple traces in a circle of this diameter, the tracks were designed in a zig-zag manner (Fig. 1C).

Apart from increasing the trace resistance (due to the increase in length), this design also reduced magnetic interference due to mutual inductance in parallel traces. The total track length was approximately 120 mm, equating to a resistance of 3.82 Ω at 25 °C given a copper thickness of 35 μm. However, it is important to note here that the track spacing was not always consistent with the design specifications and that there can be variations observed in the copper thickness, therefore the real resistance could be slightly higher than the predicted resistance. To both power and measure the resistance of the traces, a 4-wire input was provided, with the power traces having a thickness of 60 mil (1.548 mm). Measurement traces lead to a high input impedance path of an operational amplifier. The heater PCB was connected to the main PCB through a PCIe connector (the pins were reassigned for this application).

#### Electrochemistry slide

The electrochemistry PCB was designed with dimensions similar to the heater PCB, with a sample placement well that measures 7 mm in diameter (Fig. 1C). The electrochemistry PCB was also designed to connect to the main PCB via a PCIe connector. The main PCB unit and electrochemistry PCB interacted through the counter, pseudo-reference, and working electrode signals. In this PCB, the electrode dimensions are 0.25 mm^2^ for the working electrode, and 0.97 mm^2^ for the pseudo-reference electrode. and 1.34 mm^2^ for the counter electrode (see Figure S4 for CAD drawings).

#### Addition of PDMS chambers

Both the heater and electrochemistry PCBs slides needed to be fused with molded PDMS chambers with inserted plastic lids to form an all-enclosing container for the liquids involved and to avoid evaporation during the amplification. PDMS was chosen as the material since it would allow for easy integration of microfluidic functions in future iterations of the system. PDMS is conventionally fused to silica surfaces using plasma etching. However, this is not applicable for solder mask-covered FR4 surfaces. Our adhesion method was adapted from the work of Hamzah et al.^52^.

Before the addition of the PDMS chamber, the slides were cleaned as follows: first, the PCBs were immersed for 15 min in a 5:1:1 mix of MilliQ water, 30% hydrogen peroxide, and 0.5 M potassium hydroxide, respectively; then, the PCBs were ultrasonicated while immersed in ethanol for 5 min and then in MilliQ water for an additional 5 min; finally, the PCB slides were air-dried.

For molding the PDMS chambers, we used a 3D-printed mold with two open sides that were covered with thermostable tape as removable walls for easier demolding. We mixed PDMS base and curing agent solutions in a 10:1 ratio. After the PDMS mixture was poured into the molds, it was degassed in a vacuum chamber and then cured overnight at 75 °C. The PDMS was de-molded, rinsed with MilliQ, and air-dried before they were attached to the PCB slides. Liquid PDMS mixture was coated on the bottoms of a 12 cm petri dish at 300 rpm for 300 s in a EZ4 Spin Coater (Lebo Science, China). The bottom of the PDMS pieces were then stamped on the coated surface and placed on top of the PCB slides. For easier alignment, we used a simple 3D-printed mold with four pillars that pass through the holes in the PCB slide and fit within the indents in the corners of each PDMS piece (Figure S5). This allowed the central hole of the PDMS pieces to align perfectly with the circular heater or electrode regions. The liquid PDMS was cured overnight at 75 °C while the molded PDMS pieces were pressed down with a weight. Finally, a plastic lid made by cutting the top third of a standard 0.5 mL microcentrifuge tube was mounted within the top of the centrifugal hole of the PDMS chamber.

### Software

All the files for the software and hardware design are in an Open Access GitHub link^53^. The software developed to control this device has two parts: a firmware running directly on the microcontroller and a computer application for user interfacing and data acquisition. The firmware interacts with the amplifiers, heaters, and electrodes. The application software communicates with the microcontroller firmware and provides it with commands to start, change reference temperature, change electrode potential, etc. The firmware was developed in C using the STM32CubeIDE version 1.16.0. The application software was developed using MATLAB Simulink. The low-level software is designed to run non-stop on the microcontroller. The high-level software is run by the user on demand. The low-level and high-level software communicate over the Universal Asynchronous Receiver Transmitter (UART) protocol over USB for connection to computers.

### Synthetic RNA

The synthetic RNA fragments of SARS-CoV-2 and Influenza A used for the RT-LAMP were generated by in vitro T7 transcription as previously described by Tayyab et al.^54^ For the full sequence, see Table**S1**. The SARS-CoV-2 target RNA was used for the calibration curves, while the non-target Influenza A RNA was used for cross-reactivity experiments.

### Nucleic acid amplification

The RT-LAMP reaction mixture was prepared using the WarmStart LAMP kit according to the manufacturer’s instructions, but with twice the recommended volume. For each reaction, 5 µL of primer mix containing 2 µM F3, 2 µM B3 primers, 16 µM FIP primer, 16 µM BIP primer, 4 µM Loop F primer, and 4 µM Loop B primer in nuclease-free water were mixed with 25 µL LAMP master mix solution, 18 µL nuclease-free water, and 2 µL of target RNA (1-10^5^ copies per reaction), non-target RNA (10^5^ copies per reaction) or nuclease-free water (negative). When heater PCB slides were used, the reaction mix was covered with 50 µL mineral oil. The amplification was always done for 60 min at 65 °C, either loaded on a PCB slide with our main unit and its software, in a lab oven, or in a conventional PCR tube in a thermocycler (CFX96 C1000 Touch, Bio-Rad Laboratories, Hercules, CA, USA). The amplification was verified through gel electrophoresis on a 2% agarose gel using GelRed stain with flanking ladders and visualized on a GelDoc XR+ (Bio-Rad Laboratories, Hercules, CA, USA) using the accompanying ImageLab software. The LAMP products were transferred to tubes and stored in a −20 °C freezer until further use.

### Electrochemical detection

The LAMP product was mixed with a solution of Methylene Blue (MB) in Na_2_SO_4_ for a final concentration of 200 µM MB and 62.5 mM Na_2_SO_4_. This mixture was added to the electrochemistry PCB slide for electroanalytical detection. The PCB slide was inserted in the corresponding slot on the main device and connected to a computer and Cyclic Voltammetry (CV) measurements were performed with our software and the potentiostat circuit on the device. Alternatively, the device was connected to Autolab PGSTAT204N with MUX 16 module (Metrohm Autolab, Sweden) and the CVs were obtained using the accompanying NOVA 2.1.4 software package. The scan window was − 0.40 V to 0 V and the scan rate was 30 mV/s. The whole assay was performed at RT. Statistical analyses were conducted using unpaired Student’s *t*-test through SigmaStat Software, considering a *p*-value ≤ 0.05.

## Results and discussion

### Design of the lab-on-PCB system

The PCB system that we have designed consists of three main components: the main unit and two sample testing PCBs, namely a heater PCB slide and an electrochemistry PCB slide. The main unit is responsible for delivering power and processing signals from the sample testing PCBs (see Fig. 1G for a visual overview of the circuit schematic). The main unit is highly compact, with a footprint of 6.5 × 6 cm, and its dimensions could reasonably be further reduced. Combined with its low weight, the system could readily be made portable, or even handheld, rendering it well-suited for many point-of-care (POC) applications, in line with the established REASSURED criteria defined by WHO. This is particularly important for POC devices intended for use in remote or resource-limited settings, or to alleviate pressure on overstressed centralized healthcare systems, e.g. during a pandemic.

Both PCB slides (Fig. 1A) can be connected to the main unit via a standard edge connector (PCIe) (Fig. 1E). Using edge connectors has the advantage that no additional components are needed on the disposable PCB slides, as uncovered metal tracks on the PCB are directly used for making the contact. The heater PCB consists of a small integrated coil used to deliver heat to the sample for performing isothermal nucleic acid amplification and is also used in parallel to infer the temperature of the sample through the resistance of the coil. The electrochemistry PCB slide is used for electrochemical detection and consists of three electrodes arranged in a concentric circular pattern (Fig. 1C). Both PCB slides were post-modified by attachment of a molded PDMS chamber with a plastic lid, to contain the sample mix (see Fig. 1B for photographs and Fig. 1D for a schematic).

Heating elements for LAMP and other forms of heaters have typically consisted of a cartridge heater or nichrome-based heating elements. Most of these heating elements are permanent in the machine, and an external tube is therefore needed to contain the samples. After the amplification of the sample, the tube must be moved to an electrochemistry setup, where electrodes are either placed into the setup or the sample is placed on electrodes. Upon completion of testing, the electrodes need to be discarded or thoroughly cleaned to eliminate cross-contamination.

Our PCB-based electrodes are very advantageous for POC NAATs as their backing PCBs are cheap to produce at scale, not dissimilar to home glucometer tests. Importantly our heater contains no additional components.

The use of PCBs for heating is an inspiration drawn from 3D printers whose heated bed is used to prevent warping of the print. A similar PCB heater is also used in this work. There is limited work conducted in using PCB heaters for DNA amplification. For example, Skaltsounis et al.^55^described a PCB-based microreactor for fast DNA amplification. However, the work only described the design and thermal validation of this concept, but without demonstrating actual DNA amplification. Additionally, while this work focuses on DNA amplification, there is no detection mechanism built into the same PCB. In the case of our work, we can demonstrate that our setup can maintain and vary temperatures and detect DNA on the same main board.

### Calibration of heater function

Before using the heater function, it was necessary to calibrate the heaters and the associated measurement of voltage and current. This ensures that the feedback of the temperature measured matches the actual temperature achieved by the device. The heat produced in the heater chamber is not even and varies as a function of distance from the center of the heater coil. This is a result of having lower temperatures at the edges of the chamber. The drop in temperature can be determined through the heat equation:

2 $$\:\frac{\partial\:\text{U}}{\partial\:\text{t}}=\text{k}\frac{{\partial\:}^{2}\text{U}}{\partial\:{\text{x}}^{2}}$$.

**Fig. 2:**
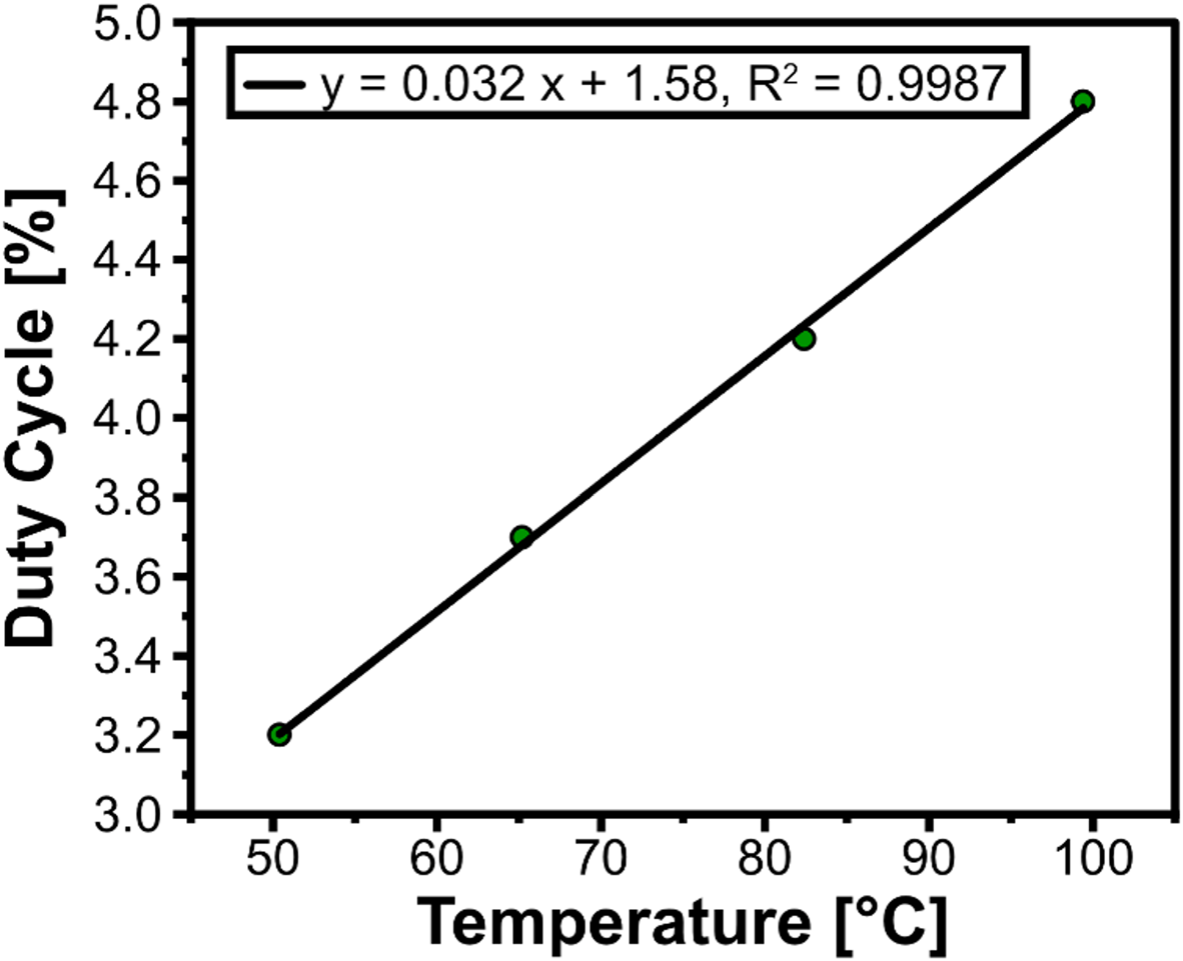
Nucleic acid amplification using the heater function. The calibration curve of the heater function of the main unit, with the percentage of the Duty Cycle plotted against the temperature.

Where *U* is the temperature on the body at the specific point in time, *t* is the time when the temperature is measured, is the thermal conductivity of the medium, and *x* is the spatial position in the chamber. The approximation with a one-dimensional heat equation can be performed since the circular cross-section has similar heat transfer when viewed in the orthogonal direction. According to the heat equation, the heat conduction varies as a result of distance. However, with time, an equilibrium is maintained. The equilibrium is disturbed when the temperature is set. Given the heat equations, for the calibration, the average temperature of the well is taken through temperature measurements. We measured the temperature for calibration using K type thermocouples. We used the RS Pro K type thermocouples with a 2 mm diameter. The readout of the thermocouple values was recorded using a custom board made of the Analog Devices AD8495 integrated circuit. Using these values, a correlation between the input PWM and temperature was determined. With this co-relation, the PWM value was adjusted to correspond to the reference temperature. This was performed as an open-loop control. The calibration curve, with the percentage of Duty Cycle plotted against the temperature, can be seen in Fig. 2.

###  Nucleic acid amplification

We chose LAMP as the amplification technique in our device because it works isothermally at a constant temperature of 60–65 °C and creates a large number of amplicons (100 times more than conventional PCR) and the relatively large size of the amplicons makes it especially suitable for our chosen electrochemical detection method (detailed below). The specific kit we used (see Sect. 2.1 Materials) also contained reverse transcriptase in the supermix for conversion of the RNA to complementary DNA (cDNA). An important disadvantage of LAMP compared to PCR is the complexity of designing suitable primers. We used an established set of primers for SARS-CoV-2 that was previously developed by Rabe et al.^56^ The primers target a non-conserved region of the SARS-CoV-2 Orf1a gene with high sensitivity and specificity (i.e. without being prone to background signals). Moreover, this set of primers is currently used with several EUA (Emergency Use Authorization) tests that detect SARS-CoV-2^57^.

To test the amplification function of our device, we loaded the LAMP reaction mixture to a heater PCB slide, covered with mineral oil to avoid evaporation and condensation away from the heating surface, and attached it to the corresponding slot on the main device. For the positive samples, the template was 10^5^ copies/µL of SARS-CoV-2 target RNA (which for the negative samples was substituted with nuclease-free water). With the computer software, we programmed the device to keep a temperature of 65 °C for 60 min. We also prepared heater PCB slides in the same way and incubated them in a lab oven at 65 °C for the same amount of time, as controls. We ran the same master mix in a conventional thermocycler with an equivalent protocol for comparison.

**Fig. 3:**
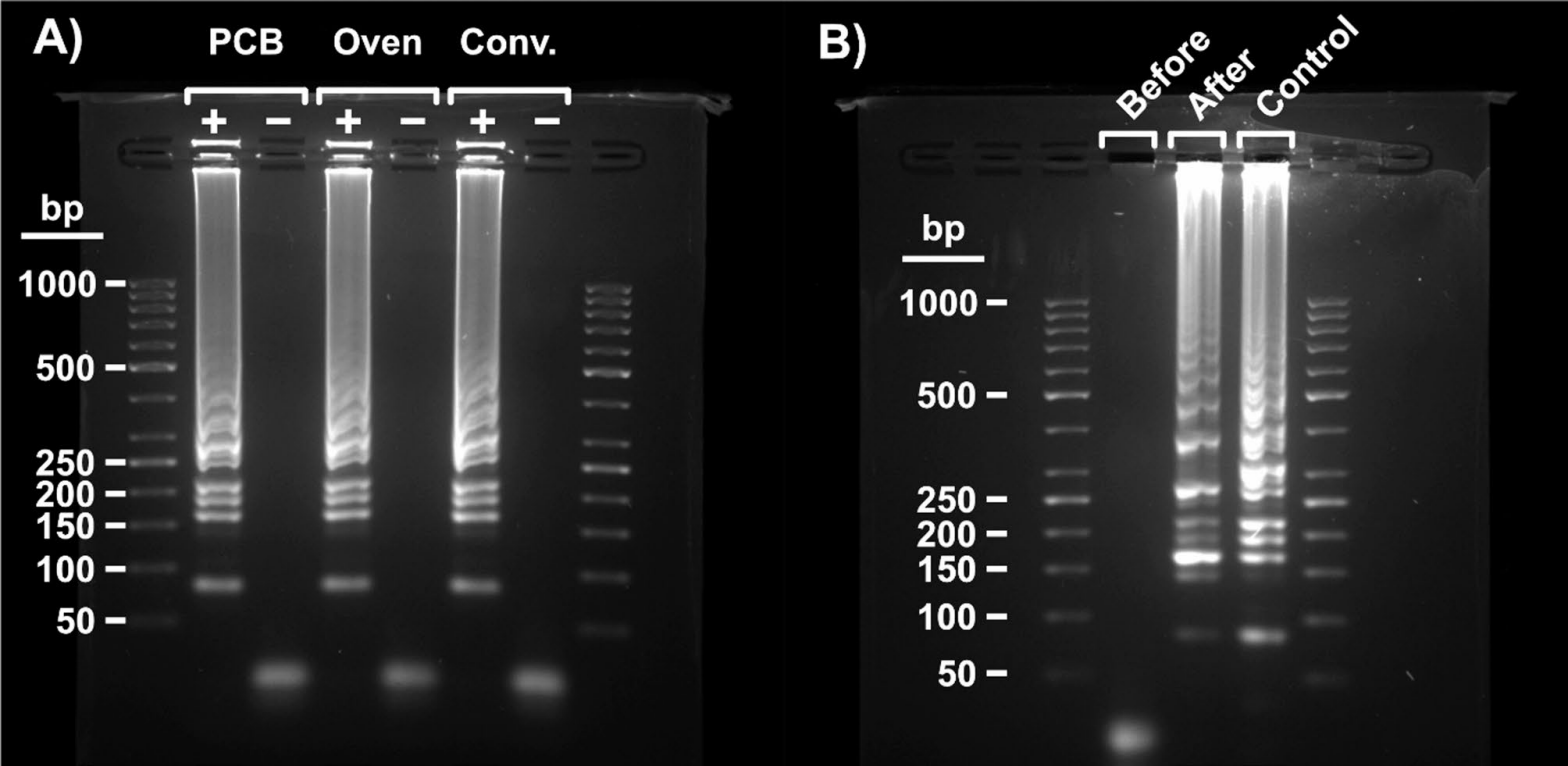
Agarose gel (2%) electrophoresis images. (**A**) Verification of successful nucleic acid amplification. Comparison of positive and negative RT-LAMP product amplified on our Lab-on-PCB device (columns 2 and 3), within a heater PCB slide in an oven (columns 4 and 5), and in a conventional thermocycler (columns 6 and 7), flanked by ladders (columns 1 and 8). (**B**) Test of enzyme deactivation protocol. Comparison of positive RT-LAMP samples when a deactivation step of 85 ºC for 10 min preceded the normal amplification protocol (column 2, marked “before”), a deactivation step was performed after the amplification (column 3, marked “after”), and no deactivation step was performed (column 4, marked “control”), flanked by ladders (columns 1 and 5). A template concentration of 10^5^ copies/reaction of synthetic SARS-CoV-2 RNA was used in all positive samples. Negative samples contained nuclease-free water.

After the LAMP reaction, the mineral oil was removed from the LAMP product and a small amount of the product was used to verify the success of the amplification through gel electrophoresis. As shown in Fig. 3, positive samples performed on PCB slides in our device have the same band pattern as those performed in an oven and a conventional thermocycler. The intensities of the bands are also similar. This indicates that LAMP reaction performed on the PCB slides is as efficient as the LAMP reaction performed using conventional approaches.

### Enzyme deactivation

While LAMP is an isothermal method and only needs a steady temperature at 65 °C, some applications with further downstream processes require enzyme deactivation at > 80 °C. Heat can also be used for bacterial and viral lysis and subsequent nucleic acid extraction^58^, as well as in sample preparation steps that exploit enzymes. While not necessary with our chosen reagents and samples, we demonstrated the potential of our device by including a function in the software that allowed us to change the temperature to 85 °C. With this, we added a deactivation step of 10 min at 85 °C at the end of the normal amplification run. In a comparative control sample, we instead put the deactivation step before the amplification run. The LAMP products were compared with an agarose gel. As observed in Fig. 3B, the deactivation was not detrimental to the LAMP product when performed after the amplification (we deem the lower intensity of some bands to be within normal variation) but completely inhibited the reaction when performed before. Due to this demonstrated ability to precisely control the temperature of the device, we believe that a future refined device could perform heat-inactivated viral lysis^58^, or other heat-dependent sample preparation steps that exploit enzymes^59–62^further delivering a fully sample-to-answer device and increasing its utility for POC applications.

### Electrochemical detection with redox-active intercalator

**Fig. 4:**
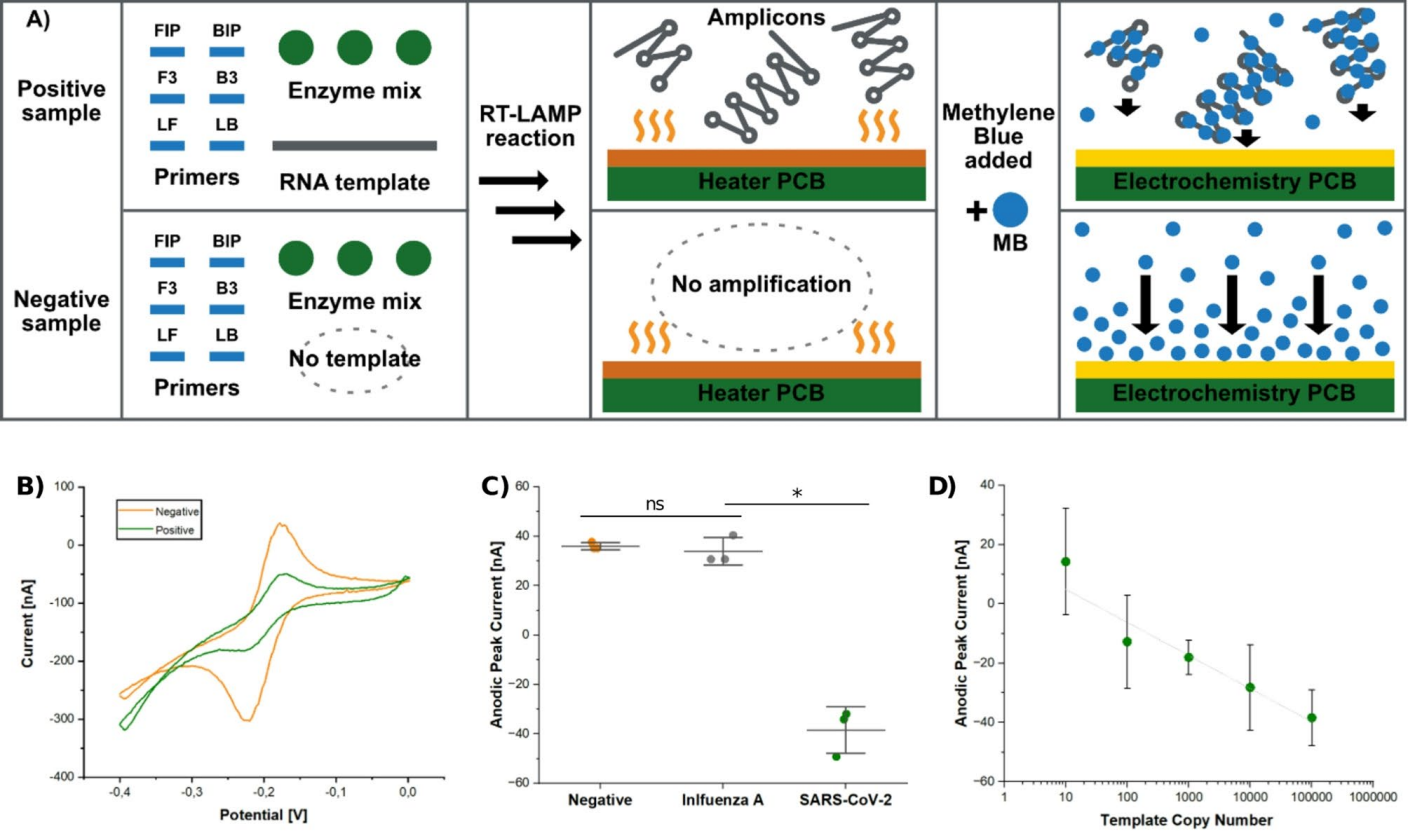
Electrochemical detection using Cyclic Voltammetry (CV) the redox-active intercalator Methylene Blue (MB). **(A)** Schematic of the detection method. In positive samples (top row), MB will intercalate with the amplicons, leading to a complex with a lower diffusion constant than unbound MB. In negative samples (bottom row), there are no such mass transport constraints. **(B)** Representative CVs of a negative (nuclease-free water) and a positive (10^5^ copies/reaction of SARS-CoV-2 RNA) using the Lab-on-PCB device, confirming the successful reduction of the anodic peak in a positive sample. **(C)** Comparison of anodic peak currents for negative (nuclease-free water), non-target RNA (10^5^ copies/reaction of Influenza A RNA), and positive (10^5^ copies/reaction of SARS-CoV-2 RNA) samples using the Lab-on-PCB device. **(D)** Calibration curve of the anodic peak current versus the template (SARS-CoV-2 synthetic RNA) copy number per reaction, using the Lab-on-PCB device. A trend line based on linear regression analysis is included. *n* = 3 for all conditions, mean with SD, unpaired *t*-test, *p*-value ≤ 0.05.

The electrochemical detection method we used was chosen for its simplicity, as it requires no functionalization or washing steps (see Fig. 4A for a schematic of the detection method). The only step is the addition of MB to the amplified product, upon which it intercalates with the dsDNA^63–66^. After applying a potential to this mixture, it is possible to discriminate the current response between intercalated MB in successfully amplified samples from the free MB in negative samples. The change in current originates from the difference in the diffusion constant of MB in the two states: intercalated MB-DNA complexes have slower diffusion, causing a mass transfer constraint^67,68^. This method has previously been used for both electrochemical qPCR^67–71^ and electrochemical quantitative LAMP^72–74^. Other intercalating redox-active molecules have also been used in similar ways^67,75^. In our device, we did not monitor the ongoing amplification in real time, but only performed end-point discrimination between positive and negative samples, which for most diagnostic applications is a sufficient level of information.

We first verified that the electrochemistry circuit of the main unit was functional and that its performance was comparable to that of a conventional potentiostat. For these initial experiments, we mixed 200 µM of MB with a supporting electrolyte (without using RT-LAMP product) and loaded the mixture onto the electrochemistry PCB slides. We inserted the slides into the dedicated slot on the main unit and performed CVs with its electrochemistry circuit while connected to a computer and controlled with our developed software, or bypassing the circuits and connecting to a conventional potentiostat. Figure S6 illustrates the CVs obtained from both our Lab-on-PCB system and the conventional potentiostat. Both systems exhibited a peak for MB at around − 0.20 V, with comparable CV shapes and current responses. Additionally, comparable CVs were obtained using a solution of 60 mM ferricyanide in 0.1 M potassium chloride (Figure S7).

Having successfully measured MB using the Lab-on-PCB system, we proceeded to analyze RT-LAMP products. In the first experimental series, we performed RT-LAMP on negative samples (nuclease-free water) and positive samples containing 10^5^ copies/reaction of SARS-CoV-2 RNA, for which representative CVs are illustrated in Fig. 4B. Negative samples showed an anodic peak of 35 nA, while positive samples showed a reduction of the anodic peak current down to −50 nA. This reduction of the anodic peak current in positive samples indicated that the redox peak from free MB was quenched with the presence of RT-LAMP product. To test the specificity of the system, RT-LAMP was performed using non-target controls containing 10^5^ copies/reaction of Influenza A RNA. As can be seen in Fig. 4C, RT-LAMP products containing either nuclease-free water or Influenza A RNA showed no statistically significant differences in anodic peaks currents, indicating no cross-reactivity with a common respiratory virus. Additionally, we analyzed the same samples with a conventional potentiostat and obtained comparable results (Figure S8).

In the second experimental series, we conducted RT-LAMP with serially diluted SARS-CoV-2 RNA, ranging from 10 to 10^5^ copies/reaction, followed by electrochemical detection using the Lab-on-PCB system. When plotting the anodic peak currents against the template copy number per reaction (Fig. 4D), a linear relationship (Y = 1.60E-08–1.12E-08X; R^2^ = 0.94) was obtained, which was also observed with the conventional potentiostat (Figure S9). This linearity indicates the potential for quantitative analysis. It is important to highlight that samples containing 10 copies/reaction could be distinguished from negative samples, indicating that the LOD of the system was as low as 10 copies/reaction. Given that symptom onset for COVID-19 typically correlates with a viral load of approximately 10^2^copies/reaction^76^, our system could detect viral presence before symptoms appear, similarly as the gold standard techniques PCR. However, further analysis of clinical samples would be needed to confirm the clinical sensitivity and specificity of the system.

We have developed a device that exploits PCB slides for nucleic acid amplification and detection, and further integrates the necessary electrochemistry circuit for heating and electrochemical measurements in a PCB. As a proof of concept, our Lab-on-PCB system successfully detected SARS-CoV-2, achieving clinically relevant analytical specificity and sensitivity and positioning it as a viable option for POC or in-field applications. However, the current design requires a manual step to transfer the product solution between chambers, which introduces potential contamination risks. Future work will focus on achieving amplification and detection at the same time or in the same chamber.

One approach to achieve this could involve integrating microfluidic channels that connect the chambers, which would also enable flushing and cleaning for sample preparation steps. Alternatively, we could combine the two functions of the PCB slides (heater and electrochemical readout) into a single PCB in a future iteration of the device by developing electrochemical quantitative RT-LAMP. Although this approach might require lower MB concentrations^72–74^, electrochemistry PCB slides could be adjusted by increasing the working electrode area and generate higher currents. Finally, future work will explore the incorporation of more sensitive electroanalytical techniques than CV (e.g., square wave voltammetry) in the Lab-on-PCB device.

## Conclusion

We have developed a Lab-on-PCB device that is designed to perform isothermal amplification of SARS-CoV-2 RNA using RT-LAMP and perform electrochemical detection using CV with an intercalating redox-active molecule. The system consists of a main unit that performs the two functions on two separate PCB slides that slot into the device. We performed successful amplification down to 10 copies of synthetic SARS-CoV-2 RNA template and demonstrated no cross-reactivity with other respiratory viruses such as Influenza A, which positions the developed system with clinically relevant analytical specificity and sensitivity. Moreover, we also adjusted the temperature of the heater to perform enzymatic deactivation, indicating the potential of using the device for heat-dependent sample preparation or downstream processes. In future iterations, it would be possible to combine the heater and electrochemical readout onto a single PCB disposable strip to allow for DNA amplification with real-time detection.

This work is an important step toward realizing a low-cost highly portable device with integrated isothermal amplification and electroanalytical detection for POC applications, towards the democratization of sample-to-answer DNA diagnostics with direct digital read-out.

## Electronic supplementary material

Below is the link to the electronic supplementary material.

Supplementary Material 1

## Data Availability

The datasets used and/or analysed during the current study available from the corresponding author on reasonable request.
