# Supplementary material for: Lab-on-PCB with integrated DNA amplification and electroanalytical detection for point-of-care diagnostics
Source: Sci Rep. 2025 Sep 12;15:32418. doi: 10.1038/s41598-025-12364-1 (PMC12432110; doi:10.1038/s41598-025-12364-1)
Supplement: Supplementary file 1 — Supplementary Material 1 [file 41598_2025_12364_MOESM1_ESM.docx]

Supplementary Information

**Lab-on-PCB with Integrated DNA Amplification and Electroanalytical Detection for Point-of-Care Diagnostics**

Martin Hanze^1+^, Seshagopalan Thorapalli Muralidharan^2+^, Alar Ainla^3^, Björn Möller^2*^, Mahiar Max Hamedi^1*^, Anna Toldrà^1,4*^

**GitHub link**

The collection of files for the software and hardware design:

<https://github.com/sesh-tech/Lab-on-PCB-with-Integrated-Amplification-and-Electroanalytical-Detection-for-Point-of-Care-DNA-Tests>

**Table S1:** Sequences of oligonucleotides used in this work.

| **Name** | **Sequence (5’-3’)** |
| --- | --- |
| As1_F3 | CGG TGG ACA AAT TGT CAC |
| As1_B3 | CTT CTC TGG ATT TAA CAC ACT T |
| As1_LF | TTA CAA GCT TAA AGA ATG TCT GAA CAC T |
| As1_LB | TTG AAT TTA GGT GAA ACA TTT GTC ACG |
| As1e_FIP | TCA GCA CAC AAA GCC AAA AAT TTA TTT TTC TGT GCA AAG GAA ATT AAG GAG |
| As1e_BIP | TAT TGG TGG AGC TAA ACT TAA AGC CTT TTC TGT ACA ATC CCT TTG AGT G |
| Target SARS-CoV-2 | cgg tgg aca aat tgt cac ctg tgc aaa gga aat taa gga gag tgt tca gac att ctt taa gct tgt aaa taa att ttt ggc ttt gtg tgc tga ctc tat cat tat tgg tgg agc taa act taa agc ctt gaa ttt agg tga aac att tgt cac gca ctc aaa ggg att gta cag aaa gtg tgt taa atc cag aga ag |
| Non-target Influenza A | gct aag aga gca att gag ctc agt gtc atc att tga aag gtt tga gat att ccc caa gac aag ttc atg gcc caa tca tga ctc gaa caa agg tgt aac ggc agc atg tcc tca agc aaa aag ctt cta caa aaa ttt aat atg gct agt taa aaa agg aaa ttc ata ccc aaa gct cag caa atc cta cat |


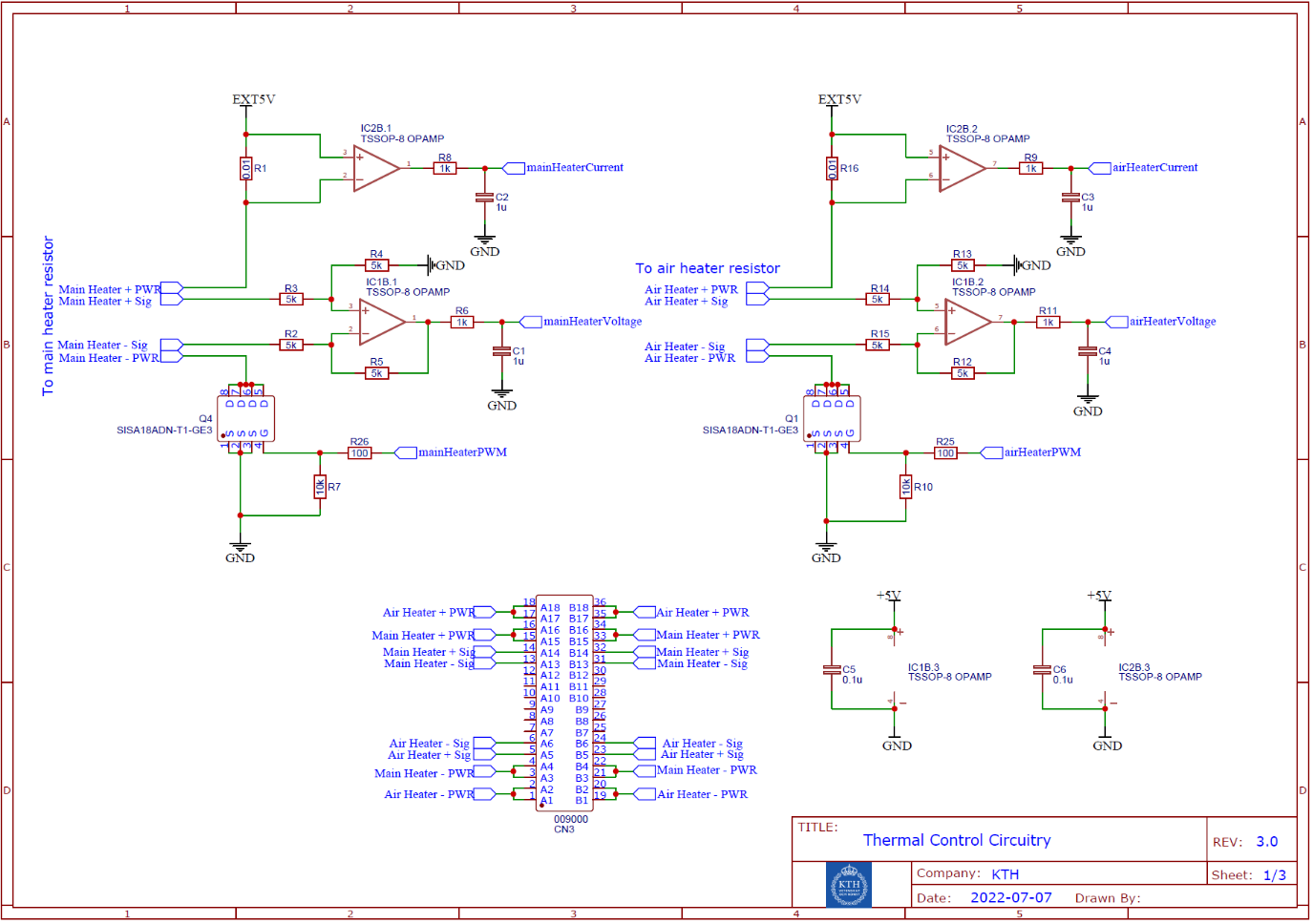


**Figure S1:** Detailed circuit schematic of the thermal control circuit of the main PCB unit.


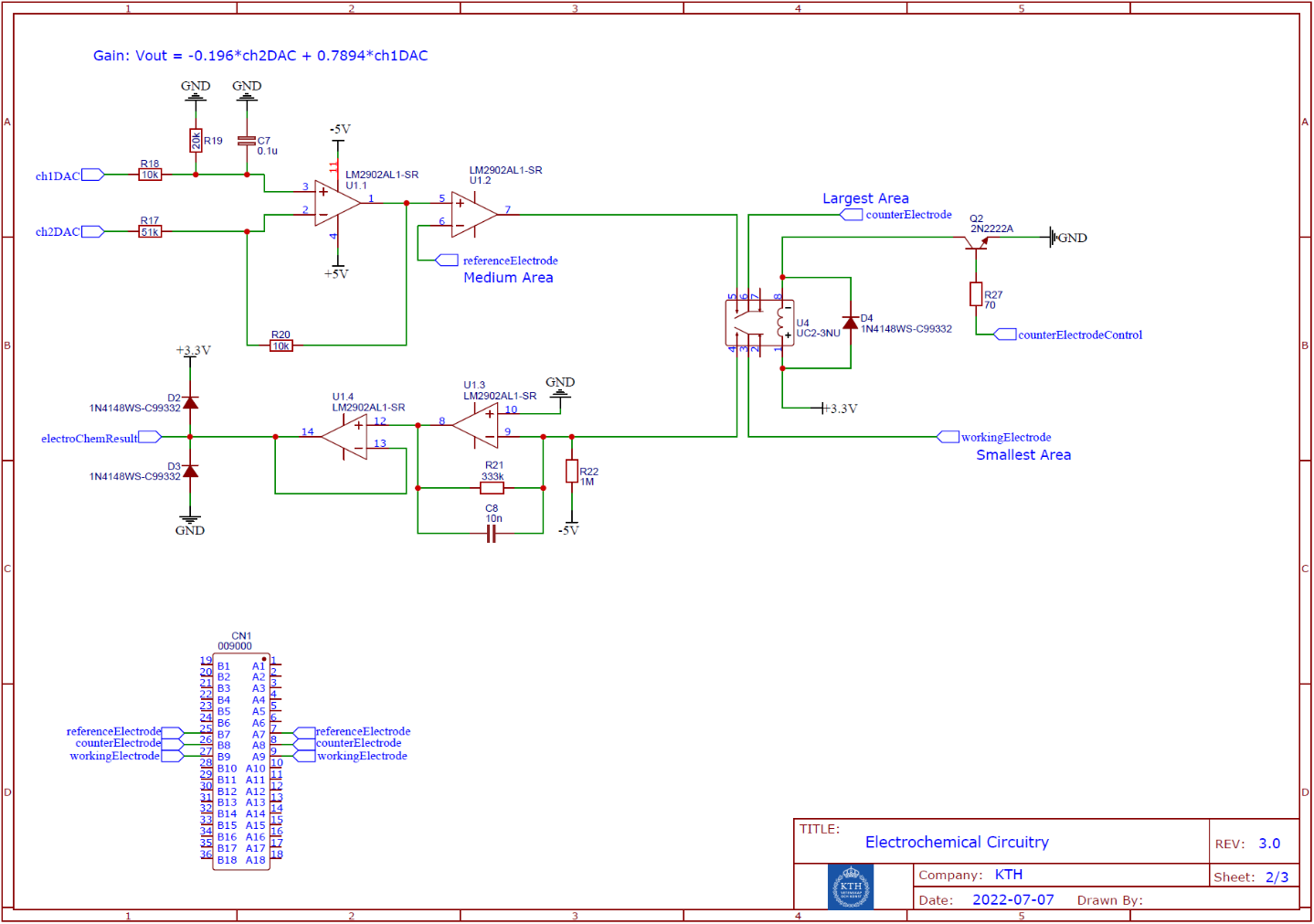


**Figure S2:** Detailed circuit schematic of the electrochemistry circuit of the main PCB unit.


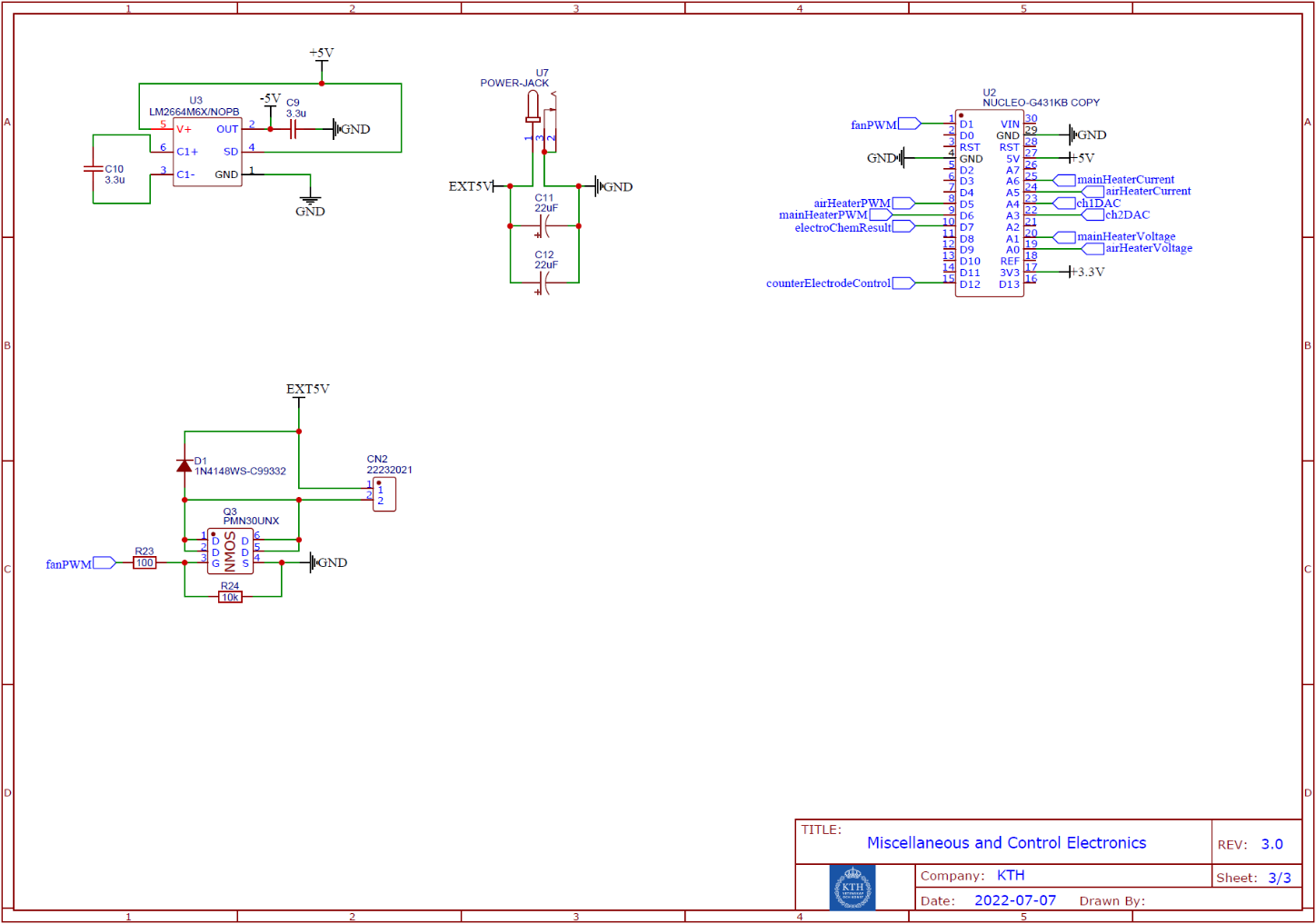


**Figure S3:** Detailed circuit schematic of miscellaneous circuits of the main PCB unit.


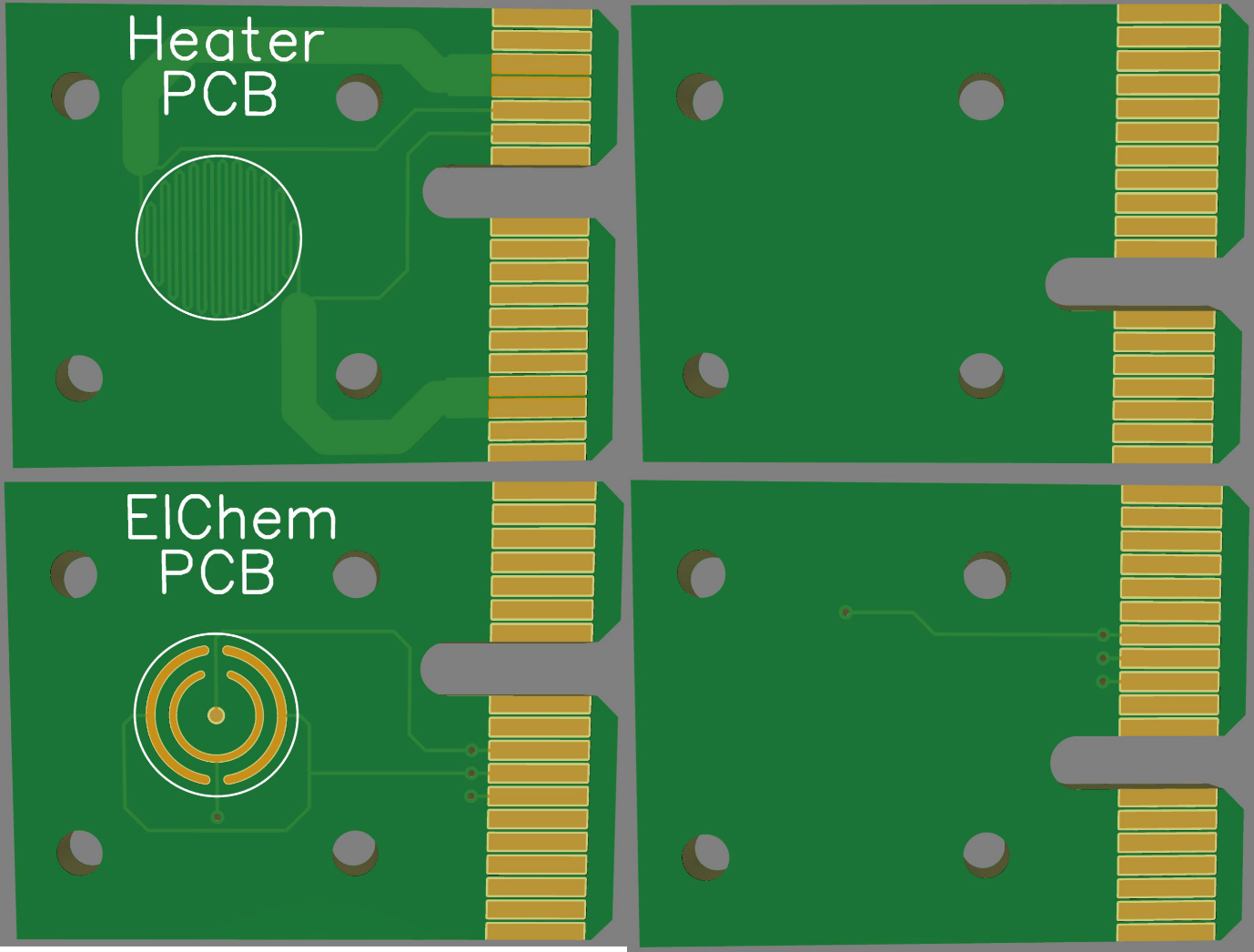


**Figure S4:** CAD drawings of the two PCB slides. The top row shows the heater PCB and the bottom row shows the electrochemistry PCB. The front sides are to the left in the figure, and the back sides are to the right.

**
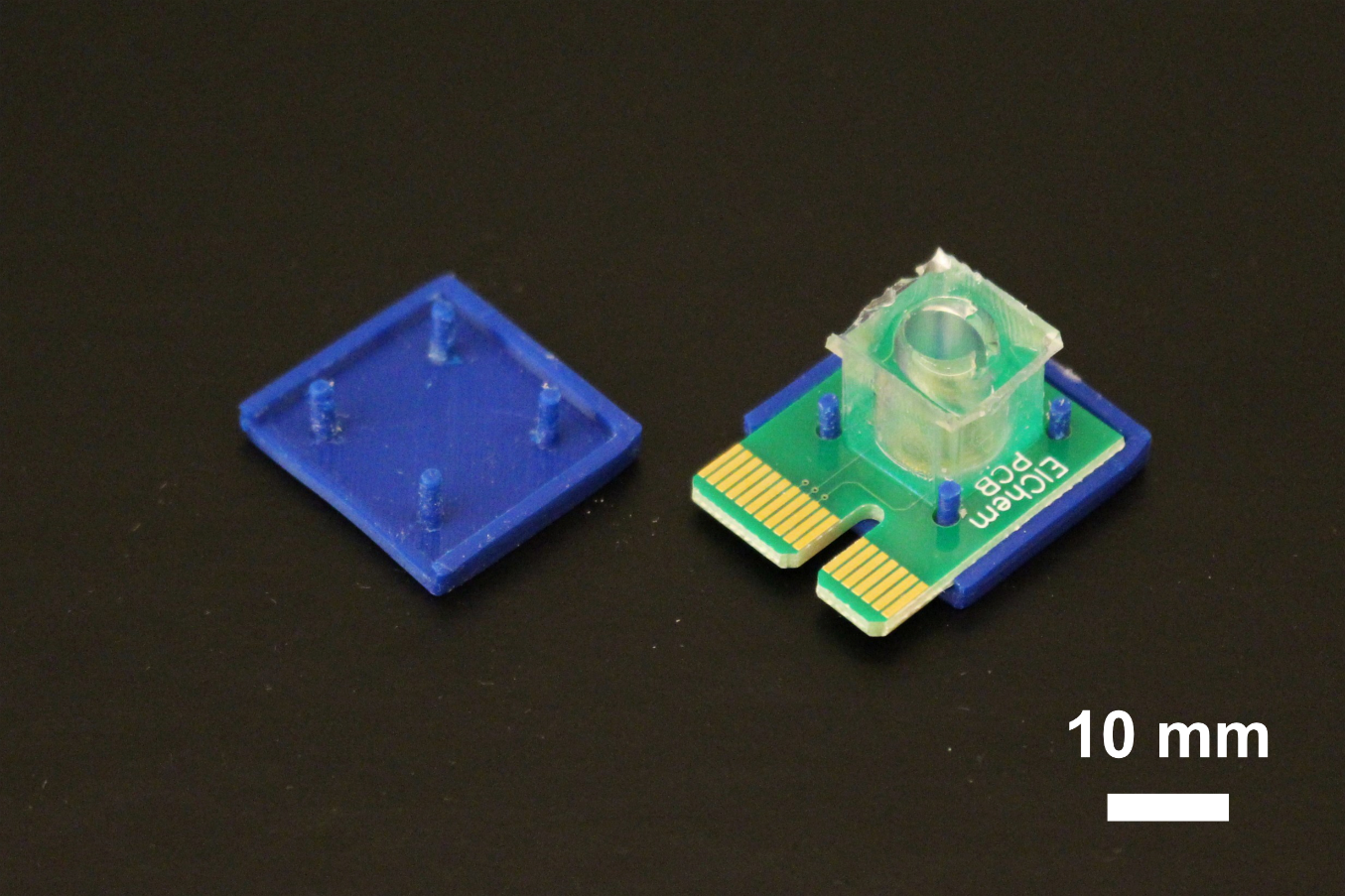
**

**Figure S5:** Photograph of the tool used for alignment when attaching the PDMS chamber. The alignment tool has four pillars that fit the holes in both PCB slides. The molded PDMS chambers have indents in the corners, which align with the pillars.


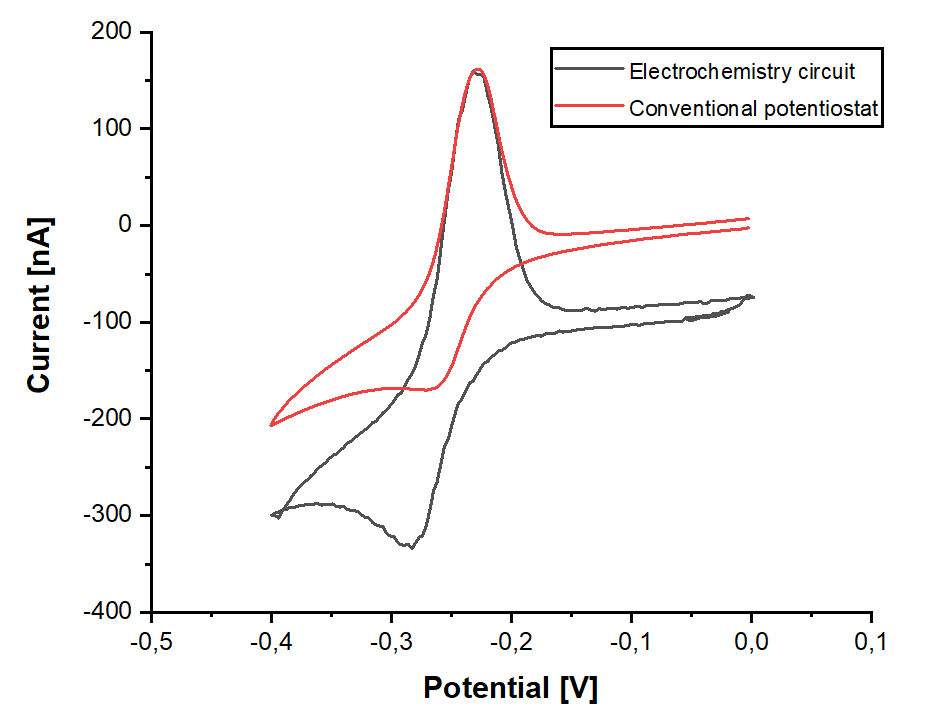


**Figure S6:** Comparison of Cyclic voltammograms (CVs) of 200 μM of Methylene Blue (MB) with a supporting electrolyte (without RT-LAMP product) that has been performed on electrochemistry PCB slides using the electrochemistry circuit of the main unit and the developed software, and with a conventional potentiostat.

**
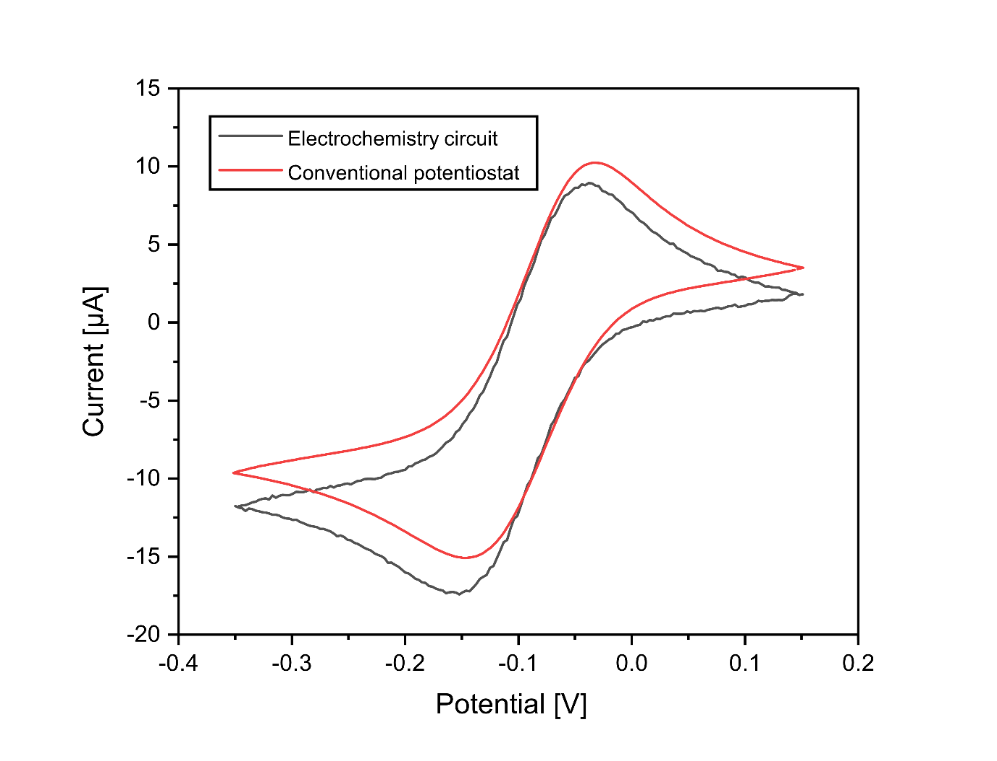
**

**Figure S7:** Comparison of Cyclic voltammograms (CVs) of 60 mM ferricyanide in 0.1 M potassium chloride that has been performed on electrochemistry PCB slides using the electrochemistry circuit of the main unit and the developed software, and with a conventional potentiostat.

**
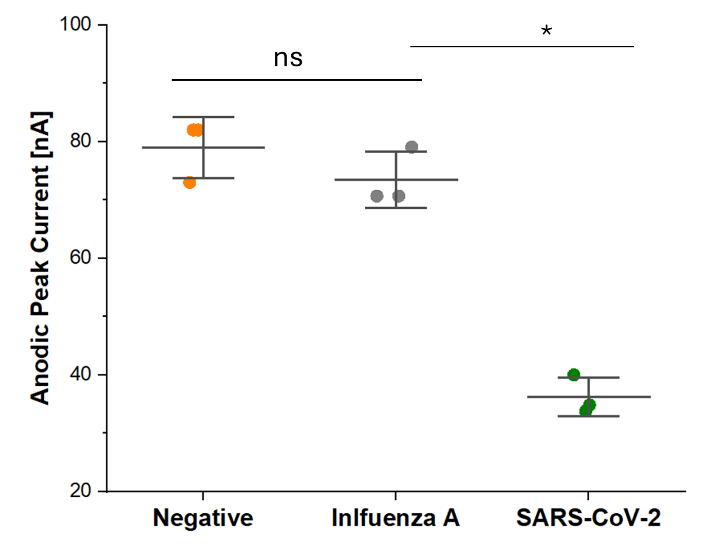
**

**Figure S8:** Comparison of anodic peak currents for negative (nuclease-free water), non-target RNA (10^5^ copies/reaction of Influenza A RNA), positive (10^5^ copies/reaction of SARS-CoV-2 RNA) samples using the conventional potentiostat. *n* = 3 for all conditions, mean with SD, unpaired *t*-test, *p*-value ≤0.05.


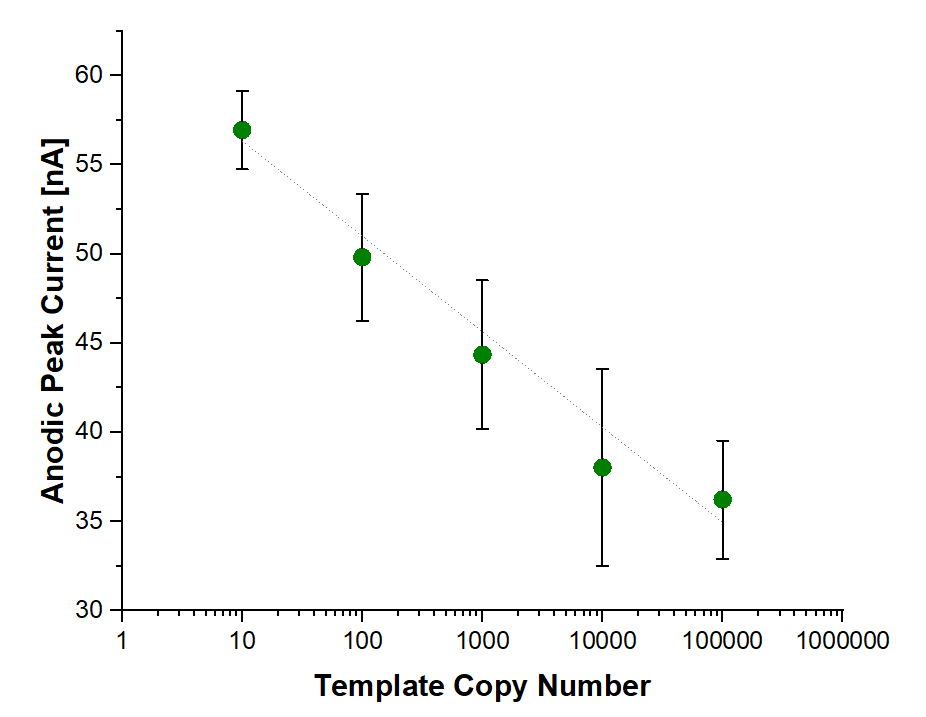


**Figure S9:** Calibration curve of the anodic peak current versus the template (SARS-CoV-2 synthetic RNA) copy number per reaction, using the conventional potentiostat. A trend line based on linear regression analysis is included. *n* = 3 for all conditions, mean with SD. Y = 6.17E-08 – 5.35E-09X; R^2^ = 0.98.
